# Amoebic Dysentery

**Published:** 1907-08

**Authors:** 


### AMOEBIC DYSTENERY.

Dr. T. A. Mitchell says under Symptoms and Diagnosis that he wishes to mention only such symptoms as appear to him to be diagnostic being found in the literature. While the diagnosis of amoebic dysentery is made absolute by finding the amoebia in the stools, it is not necessary to look for them in every case of acute dysentery, but chronic cases should all be examined for amoebia if the diarrhoea is irregular or intermittent with infrequent tenesmus and moderate fever. Chronic dysentery is afebrile and the stools are uniform in character, quanity and frequency. The cell elements are few and the amoebia are easily detected if present. The onset may be abrupt or gradual, but in most cases a frequent and painless diarrhoea follows a period of slight ill health. True relapses are common and the tendency to chronicity is very great. Diarrhoea may be the only feature of the disease. It is characterized by great variation in character and frequency in all grades and during different periods of the disease. Intermissions and exacerbations may be observed at any time. The stools are extremely variable, according to the severity of the ulceration. In the more moderate types the stools at the outset are like those of gangrenous dysentery if the attack is abrupt. If gradual the stools are fecal liquid, containing mucous and streaks of blood and gelatinous grayish masses. Stools of this character number from four to ten in twenty-fours hours. This may continue for weeks. During the exacerbations the stools resemble those of the second of gangrenous fever. In chronic dysentery there is not so much mucous and blood except in exacerbations. Mucous is persistently present, however, in the intermissions when the stools are soft and fecal. Abdominal pain is constant; it occurs in the early stages of both forms and in acute exacerbations. In the gangrenous form pain disappears, although the intensity of the process in increasing. In all cases the pain is cramp-like, boring or burning in character and usually precedes and accompanies movements of the bowels.

Treatment.—Formalin and aqueous sol. of formaldehyde gas about 40 per cent., which is equal in antiseptic and germicidal properties to bichloride of mercury and can be used in strength of from

1:1000. In a case treated twelve years ago the auther used nitrate of silver 1 dram to one-half gallon of water.—Southern Med. & Sur. April, 1907.

